# SNP Regulation of microRNA Expression and Subsequent Colon Cancer Risk

**DOI:** 10.1371/journal.pone.0143894

**Published:** 2015-12-02

**Authors:** Lila E. Mullany, Roger K. Wolff, Jennifer S. Herrick, Matthew F. Buas, Martha L. Slattery

**Affiliations:** 1 University of Utah, School of Medicine, Department of Internal Medicine, Salt Lake City, Utah, United States of America; 2 Division of Public Health Sciences, Fred Hutchinson Cancer Research Center, Seattle, Washington, United States of America; Universidade de Sao Paulo, BRAZIL

## Abstract

**Introduction:**

MicroRNAs (miRNAs) regulate messenger RNAs (mRNAs) and as such have been implicated in a variety of diseases, including cancer. MiRNAs regulate mRNAs through binding of the miRNA 5’ seed sequence (~7–8 nucleotides) to the mRNA 3’ UTRs; polymorphisms in these regions have the potential to alter miRNA-mRNA target associations. SNPs in miRNA genes as well as miRNA-target genes have been proposed to influence cancer risk through altered miRNA expression levels.

**Methods:**

MiRNA-SNPs and miRNA-target gene-SNPs were identified through the literature. We used SNPs from Genome-Wide Association Study (GWAS) data that were matched to individuals with miRNA expression data generated from an Agilent platform for colon tumor and non-tumor paired tissues. These samples were used to evaluate 327 miRNA-SNP pairs for associations between SNPs and miRNA expression levels as well as for SNP associations with colon cancer.

**Results:**

Twenty-two miRNAs expressed in non-tumor tissue were significantly different by genotype and 21 SNPs were associated with altered tumor/non-tumor differential miRNA expression across genotypes. Two miRNAs were associated with SNP genotype for both non-tumor and tumor/non-tumor differential expression. Of the 41 miRNAs significantly associated with SNPs all but seven were significantly differentially expressed in colon tumor tissue. Two of the 41 SNPs significantly associated with miRNA expression levels were associated with colon cancer risk: rs8176318 (*BRCA1*), OR_AA_ 1.31 95% CI 1.01, 1.78, and rs8905 (*PRKAR1A*), OR_GG_ 2.31 95% CI 1.11, 4.77.

**Conclusion:**

Of the 327 SNPs identified in the literature as being important because of their potential regulation of miRNA expression levels, 12.5% had statistically significantly associations with miRNA expression. However, only two of these SNPs were significantly associated with colon cancer.

## Introduction

MicroRNAs (miRNAs) are small, non-coding regulatory RNA molecules [1–5] that have the ability to alter gene expression and thus biological pathway function. MiRNAs have been associated with several diseases, including colorectal cancer risk and survival [6–8]. The importance of miRNAs in the carcinogenic process is under intense study. It has been proposed that SNPs located in miRNA gene loci and shown to be associated with colorectal cancer may be operating through their influence on miRNA expression levels [9]. Others have proposed that SNPs within miRNA target genes (i.e. mRNAs) can influence miRNA binding of these genes through the alteration of miRNA binding sites within the mRNA, causing altered expression of mRNA [10]. Mature miRNA levels have been proposed to be correlated with the presence of their target mRNAs *in vitro* within model organisms [11], and as such, SNPs within target genes could alter miRNA expression due to altered mRNA levels. Thus, it also has been proposed that SNPs within miRNA target genes (mRNAs) that are associated with disease risk could be operating through their influence on miRNAs. The majority of miRNA regulation occurs through the binding of the miRNA seed region (~7–8 nucleotides at the 5’ UTR end of the miRNA) to the 3’ UTR of the mRNA [12]; as such, SNPs within the 3’ UTR of miRNA-target genes may create or destroy miRNA binding sites [10], and are of particular interest to this investigation.

We are in a unique position to assess whether SNPs in miRNA gene loci and SNPs in miRNA target genes influence miRNA expression in colon tissue, and in turn investigate whether these same variants influence risk of colon cancer. To test the hypothesis that SNPs alter miRNA expression levels, we use non-tumor tissue and evaluate differences in expression across genotype. However, since a SNP could alter miRNA expression levels equally in both tumor and non-tumor tissue, such an association would not necessarily contribute to cancer risk. To determine if SNPs are associated with the carcinogenic process through miRNA regulation, we also evaluate if miRNA expression is different across genotypes between tumor and non-tumor tissues. Additionally, we assess if miRNAs that are associated with SNPs are differentially expressed in colon tumors. Finally, we evaluate the association between SNPs associated with miRNA expression and risk of colon cancer.

## Methods

### Study population

The study population consisted of individuals previously enrolled in a study of Diet, Lifestyle, and Colon cancer at the University of Utah and the Kaiser Permanente Medical Research Program (KPMRP) [13] for whom GWAS data as well as miRNA from both tumor and non-tumor tissue was available, and at the Twin Cities metropolitan area in Minnesota for GWAS data only (Table 1). Study subjects included incident cases of colon cancer between the ages of 30 and 79 who were non-Hispanic white, Hispanic, or African American, and were able to provide a signed informed consent prior to participation in the study. The study was approved by the University of Utah Institutional Review Board for Human Subjects.

**Table 1:** Descriptive table of included subjects.

	GWAS				miRNA		GWAS and miRNA	
	Controls		Cases		Cases		Cases	
	N	%	N	%	N	%	N	%
**Sex**								
*Male*	643	54.8	618	55.4	606	52.7	193	56.1
*Female*	De	45.2	497	44.6	544	47.3	151	43.9
**Center**								
*Kaiser*	290	24.7	356	31.9	585	50.9	191	55.5
*Minn*.	609	51.9	527	47.3	0	0.0	0	0.0
*Utah*	274	23.4	232	20.8	565	49.1	153	44.5
**Stage**								
*I*	0	0.0	346	34.7	296	27.9	90	26.4
*II*	0	0.0	291	29.2	286	27.0	103	30.2
*III*	0	0.0	270	27.1	283	26.7	115	33.7
*IV*	0	0.0	91	9.1	195	18.4	33	9.7
**Site**								
*Proximal*	0	0.0	505	49.1	557	49.1	176	51.2
*Distal*	0	0.0	523	50.9	578	50.9	168	48.8
**Age**								
	**Mean**	**SD**	**Mean**	**SD**	**Mean**	**SD**	**Mean**	**SD**
	65.3	9.9	65.0	9.9	65.4	9.5	65.8	9.0

### miRNA processing

RNA (miRNA) was extracted from formalin-fixed paraffin embedded tissues. We assessed slides and tumor blocks that were prepared over the duration of the study prior to the time of miRNA isolation to determine their suitability. The study pathologist reviewed slides to delineate tumor and non-tumor tissue. Cells were dissected from 1–4 sequential sections on aniline blue stained slides using an H&E slide for reference. Total RNA containing miRNA was extracted, isolated, and purified using the RecoverAll Total Nucleic Acid isolation kit (Ambion); RNA yields were determined using a NanoDrop spectrophotometer. 100 ng total RNA was labeled with Cy3 and hybridized to Agilent Human miRNA Microarray V19.0 and were scanned on an Agilent SureScan microarray scanner model G2600D. The Agilent Human microarray was generated using known miRNA sequence information compiled in the Sanger miRBase database v19.0. The microarray contains probes for 2006 unique human miRNAs, with one to four unique probes for each of the known miRNAs. The miRNA array contains 60,000 unique human sequences and averages 30 replicates per probe sequence. Data were extracted from the scanned image using Agilent Feature Extract software v.11.5.1.1. Data were required to pass stringent QC parameters established by Agilent that included tests for excessive background fluorescence, excessive variation among probe sequence replicates on the array, and measures of the total gene signal on the array to assess low signal. If samples failed to meet quality standards for any of these parameters, the sample was re-labeled, hybridized to arrays, and scanned. If a sample failed QC assessment a second time the sample was deemed to be of poor quality and the individual was excluded from down-stream analysis. To minimize differences that could be attributed to the array, amount of RNA, location on array, or other factors that could erroneously influence expression, total gene signal is normalized by multiplying each sample by a scaling factor stratified by tumor site. Within each tumor site, the scaling factor [14] (http://genespring-support.com/files/gs_12_6/GeneSpring-manual.pdf) is the median of the 75^th^ percentiles of all the samples divided by the individual 75^th^ percentile of each sample.

We refer to miRNAs using standard nomenclature used in the miRBase database [15]. In this notation, the first three letters signifies the organism, and these are followed by a unique number. The number is followed by a dash and number (i.e., −1) if more than one loci codes for the miRNA. A lettered suffix denotes closely related miRNAs. If two miRNAs are coded by the same precursor product then the minor product is assigned the suffix (*). If predominant/minor product status is not known then the suffix −5p and −3p are used to denote 5′ and 3′ arm respectively. One example would be, let-7 may be reported in the literature previously, however since then let-7 has been further delineated to several closely related mature sequences and genomic loci, including let-7a-3p, let-7a-5p, and let-7b-3p.

We previously tested the reliability of the Agilent Microarray over time, repeating eight tumor and five matched normal samples (for a total of 13 samples) that had be scanned over the course of the study, and found repeatability correlation coefficient of 0.98 [16]; previous comparison of Agilent miRNA expression for select miRNAs to that generated by qPCR showed 100% agreement in terms of both direction of change and fold change [17].

### GWAS

Blood was drawn for participants who provided informed consent; these participants included those from the aforementioned study at the University of Utah, KPMRP, and in the Twin Cities metropolitan area in Minnesota [13]. GWAS data were obtained using Illumina HumanHap 550K, 610K as part of the GECCO study and has been described previously [18]. Imputation to HapMap2 Release 24 was performed using MACH which was imputed to HapMap Release 22 using BEAGLE. GWAS samples obtained from Utah and Minnesota are being uploaded in NCBI’s dbGaP (http://www.ncbi.nlm.nih.gov/gap) by the Fred Hutchinson’s Cancer Research Center.

### Selection of miRNA-related SNPs

A literature search was conducted to identify all miRNA-related SNPs shown to be associated with susceptibility to any type of cancer and more specifically to colorectal cancer [9,19–51]. We excluded SNPs from consideration that failed Illumina quality measures or standard quality control procedures [52]. Specifically, SNPs were excluded if any of the following criteria were satisfied: i) Illumina GenTrain score < 0.6 or cluster separation < 0.4; ii) discordant genotype calls in any pair of duplicate study samples; iii) Mendelian error in either one of the HapMap QC trios or a small number of families identified in the BEACON data; iv) significant departure from Hardy-Weinberg Equilibrium (P<10^4^).

### Statistical Analysis

**Fig 1:**
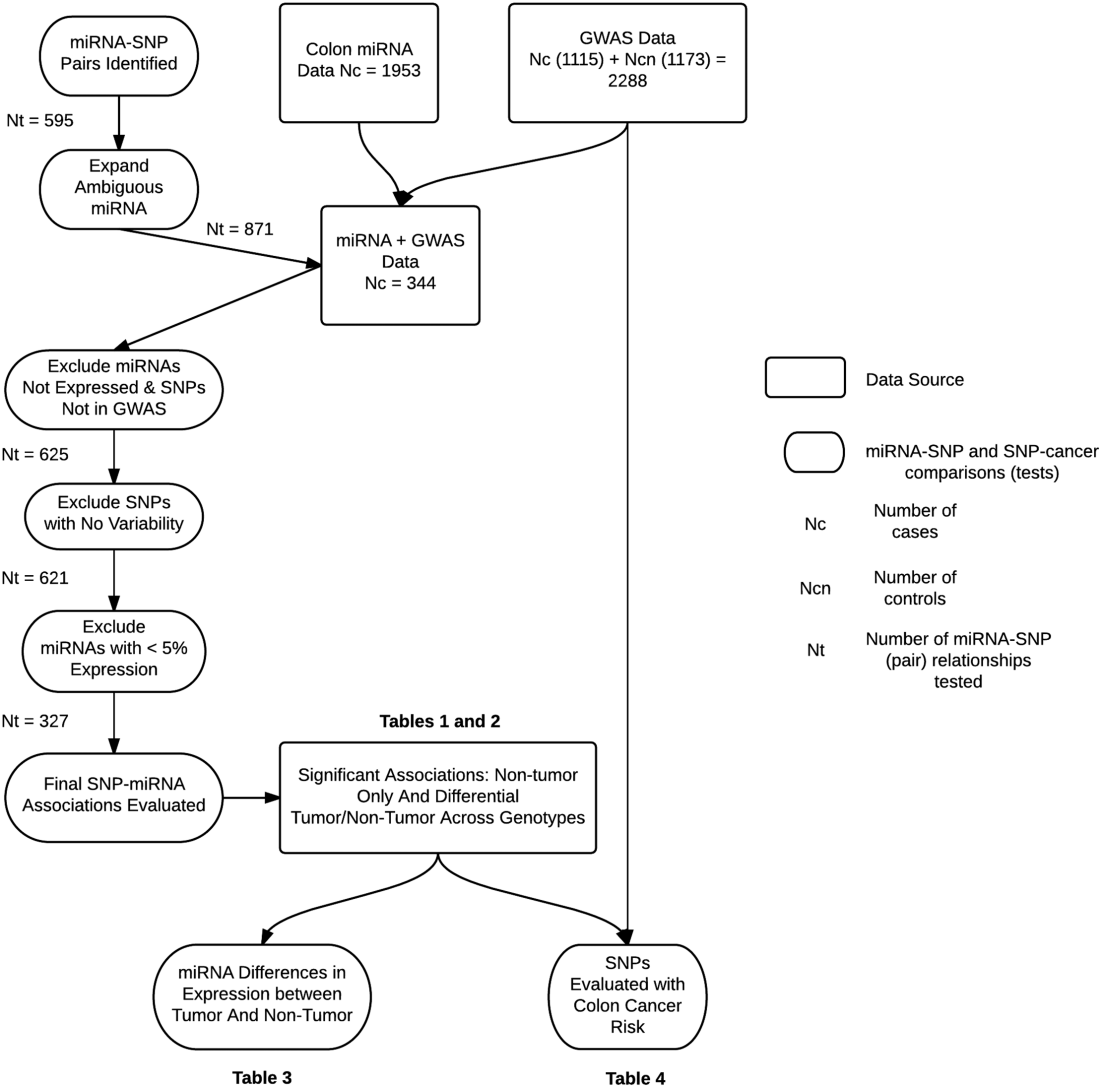
Illustration of the study flow.

The study data flow is shown in Fig 1 and illustrates the sequence leading to the final analysis of miRNAs and their related SNP. Our sample consisted of 344 subjects who had both miRNA data and GWAS data and 1,115 cases and 1,173 controls with SNP data for colon risk assessment. We started with a total 559 miRNA-SNP pairs. These pairs were expanded to encompass specific miRNA terminology (i.e. 3p, 5p, etc) to yield 835 miRNA-SNP pairs for evaluation. From this number, we excluded miRNAs that were not expressed in at least one colon sample leaving 622 pairs. We further excluded any SNPs for which we did not have GWAS information, leaving 552 pairs. We also excluded SNPs that did not have variation (i.e. few with minor allele in our sample), leaving 548 pairs. Our final analysis included 327 pairs after we further excluded any miRNAs, and their associated SNP, that did not have expression in non-tumor colon tissue in at least 5% of the population.

We utilized various bioinformatics tools. DbSNP (http://www.ncbi.nlm.nih.gov/projects/SNP/) was utilized to identify SNP coordinates: all SNP chromosomal coordinates are from the assembly GRCh37 (GRCh37.p13). Ensembl’s Variant Effect Predictor (VEP) tool was utilized through the Ensembl GRCh37 site (http://grch37.ensembl.org/info/docs/tools/vep/index.html) to yield variant location and effect. VEP was utilized using all default settings. All SNPs were found in VEP. MiRNASNP v2.0, based on miRBase build 19 and dbSNP version 137 (http://bioinfo.life.hust.edu.cn/miRNASNP2/index.php) [53] was utilized to identify predicted lost and gained regulating miRNAs as a result of 3’ UTR changes in mRNA genes due to SNPs associated with colon cancer risk. These miRNAs were then also evaluated with their associated SNPs for significant alterations in miRNA expression.

We evaluated miRNA expression in both non-tumor colonic tissue and expression differences between tumor and non-tumor tissue to obtain a better understanding of how the SNPs relate to miRNA expression. Looking at non-tumor tissue allowed us to evaluate the effect of the SNP on miRNA expression. A SNP associated with miRNA expression across genotypes in non-tumor tissue could influence the baseline expression level of the miRNA but not influence cancer risk if it equally altered miRNA expression in tumors. Thus, changes in expression between tumor and non-tumor tissue were evaluated because of their potential importance for the carcinogenic process.

Statistical analysis was performed in three stages. First, we evaluated the miRNA expression for linear trend across genotypes adjusting for age, study center and sex using a linear regression model. P-values were generated from the bootstrap method [54] by creating a distribution of 10,000 β coefficients of the genotypes and evaluating H_0_: β = 0 vs. H_1_: β≠0 using R 3.1.2 (cran.r-project.org). All statistical analysis was performed using miRNA expression data that was both normalized and log2 transformed. The mean expressions reported in Tables 2, 3 and 4 are normalized but not log2 transformed in order to present the data in a more intuitive manner. All subsequent analysis was performed in SAS 9.4 4 (SAS Institute, Cary, NC). We report crude p-values, since each paired analysis was specifically hypothesized, as well as an adjusted p-value, taking into consideration all 327 comparisons made using the false discovery rate (FDR) [55]. Secondly, we evaluated miRNAs and SNPs from previously identified pairs to determine their association with colon cancer. In this analysis, we evaluated differential miRNA expression between tumor and non-tumor colonic tissue to determine if miRNA expression was differentially expressed in colon tumors. We used a paired t-test for this analysis and calculated P-values using 10,000 bootstrap samples. Finally, we evaluated colon cancer risk associated with SNPs that significantly influenced miRNA expression using logistic regression analysis. We report Odds Ratios (OR) and 95% Confidence Intervals (CI) that have been adjusted age, sex, and study center.

**Table 2:** Associations between miRNA expression and SNPs in miRNA genes and miRNA-target genes by genotype in non-tumor tissue (adjusted for age, sex and center).

			AA			AB			BB			p-values		
Gene(s)	SNP	miRNA	N	Mean	% 0 Exp	N	Mean	% 0 Exp	N	Mean	% 0 Exp	Raw	FDR	Variant Type^3^
**miRNA**														
*CD22*, *MIR5196*	rs10406069, 0 = GG 1 = GA 2 = AA	hsa-miR-5196-5p	218	64.14	0.00	105	60.93	0.00	21	61.38	0.00	0.029	0.52	IN, DS, MS, 3' UTR, of *CD22*. NC, NCE of *MIR5196*.
*MCM7*, *MIR106B*, *MIR25*, *MIR93*	rs1527423, 0 = AA 1 = AG 2 = GG	hsa-miR-25-3p	86	14.51	9.30	182	12.40	10.44	76	10.41	21.05	0.004	0.49	DS of *COPS6*. IN, NC, DS, NMD of *MCM7*. UP of *MIR106B*, *MIR25*, *MIR93*.
*GNN*, *HSP90B1*, *MIR3652*	rs17797090, 0 = GG 1 = GA 2 = AA	hsa-miR-3652	288	159.68	0.00	56	145.86	0.00	0	NA	NA	0.032	0.52	RR. TFB, 5' UTR, NMD, NC, NCE, IN of *HSP90B1*. NCE, NC of *MIR3652*. UP of *RP11-642P15*.
*MIR146A*	rs2910164, 0 = GG 1 = GC 2 = CC	hsa-miR-146a-5p	197	8.24	32.49	129	8.29	31.01	18	6.61	5.56	0.038	0.52	NC, NCE.
*MIR1307*, *USMG5*	rs7911488, 0 = AA 1 = AG 2 = GG	hsa-miR-1307-3p	154	13.03	0.00	153	13.03	0.65	37	11.27	0.00	0.019	0.52	NC, NCE of *MIR1307*. UP of *PDCD11*. IN, 5' UTR of *USMG5*.
*RPL17*, *RPL17-C18orf32*, *SNORD58A*, *SNORD58*	rs1943676, 0 = AA 1 = AG 2 = GG	hsa-miR-1539	147	4.24	36.73	167	4.10	53.89	30	4.02	56.67	0.004	0.49	RR. UP of *C18orf32*. IN, NC, DS of *MIR1539*. SP, IN, NC, NCE, UP of RPL17. UP, DS of *SNORD58A*, *SNORD58B*, *SNORD58C*. DS of *SRP72P1*.
**miRNA-Target**														
*NRSN1*	rs1053047, 0 = GG 1 = GA 2 = AA	hsa-miR-143-5p	92	3.96	94.57	175	3.39	92.57	77	5.36	84.42	0.029	0.52	3' UTR, DS, IN.
KIAA0423 (FAM179B)*	rs1053667, 0 = TT 1 = TC 2 = CC	hsa-miR-19b-3p	305	11.19	14.10	39	13.44	28.21	0	NA	NA	0.028	0.52	DS, 3' UTR, NMD.
*SLC10A7*	rs1057560, 0 = GG 1 = GA 2 = AA	hsa-miR-25-3p	97	11.54	14.43	171	12.39	13.45	76	14.09	7.89	0.039	0.52	RR. 3' UTR, DS.
*DNM2*	rs12232826, 0 = GG 1 = GT 2 = TT	hsa-miR-638	314	3980.05	0.00	30	4412.26	0.00	0	NA	NA	0.013	0.52	IN.
*CSK*	rs1378940, 0 = AA 1 = AC 2 = CC	hsa-miR-4513	159	27.91	0.00	139	30.08	0.00	46	30.68	0.00	0.027	0.52	RR. UP, IN, NC.
		hsa-miR-1207-5p	212	2043.96	0.00	117	1911.72	0.00	15	1699.90	0.00	0.016	0.52	
*ICOS*	rs1559931, 0 = GG 1 = GA 2 = AA	hsa-miR-196b-5p	212	9.00	24.53	117	8.24	16.24	15	6.35	0.00	0.037	0.52	RR. 3' UTR.
		hsa-miR-2117	212	4.53	50.47	117	4.61	46.15	15	4.74	20.00	0.033	0.52	
*LAD1*	rs16848494, 0 = CC 1 = CT 2 = TT	hsa-miR-143-5p	328	4.28	90.85	16	0.00	100.00	0	NA	NA	0.027	0.52	DS, 3' UTR, IN of *LAD1*. UP of *TNNT2*.
*AFF1*	rs17703261, 0 = AA 1 = AT 2 = TT	hsa-miR-648	211	18.23	0.00	121	17.48	0.00	12	14.98	0.00	0.022	0.52	3' UTR, DS.
		hsa-miR-1207-5p	212	2043.96	0.00	117	1911.72	0.00	15	1699.90	0.00	0.020	0.52	
*ICOS*	rs4404254, 0 = TT 1 = TC 2 = CC	hsa-miR-196b-5p	212	9.00	24.53	117	8.24	16.24	15	6.35	0.00	0.034	0.52	3' UTR.
		hsa-miR-2117	212	4.53	50.47	117	4.61	46.15	15	4.74	20.00	0.035	0.52	
*NKAIN4*, *FLJ16779*	rs720607, 0 = GG 1 = GA 2 = AA	hsa-miR-3196	112	1415.24	0.00	163	1326.52	0.00	69	1269.88	0.00	0.027	0.52	RR. IN.
*CD80*	rs7628626, 0 = CC 1 = CA 2 = AA	hsa-miR-2117	233	4.67	42.06	98	4.40	62.24	13	3.69	38.46	0.010	0.52	RR. DS, 3' UTR, of *CD80*. DS of *TIMMDC1*.
*FGF2*	rs7683093, 0 = CC 1 = CG 2 = GG	hsa-miR-92b-3p	249	1.34	93.17	84	2.01	82.14	11	0.73	81.82	0.008	0.52	RR. DS, 3' UTR of *FGF2*. IN, NMD, DS, US of *NUDT6*.
*BRCA1*	rs8176318, 0 = CC 1 = CA 2 = AA	hsa-miR-525-5p	165	3.20	46.67	130	3.16	51.54	49	3.85	69.39	0.035	0.52	3' UTR, DS.
*PARP1*	rs8679, 0 = AA 1 = AG 2 = GG	hsa-miR-630	212	364.38	0.00	106	392.44	0.00	26	541.85	0.00	0.013	0.52	3' UTR, DS.
SELS (VIMP)*	rs9874, 0 = TT 1 = TC 2 = CC	hsa-miR-181a-5p	268	25.24	0.00	69	29.71	0.00	7	25.02	0.00	0.027	0.52	3' UTR, DS.
*MCM7*, *AP4M1*	rs999885, 0 = AA 1 = AG 2 = GG	hsa-miR-25-3p	88	14.45	9.09	180	12.36	10.00	76	10.51	22.37	0.003	0.49	IN, NMD, UP, NC, DS of *AP4M1*. UP of *MCM7*. DS of *TAF6*.

^1^Related SNPs are those in linkage disequilibrium to SNPs within the dataset.

^2^EX: Exon; DS: Downstream; IN: intron; MS: Missense; NC: Non-coding; NCE: Non-coding exon; NMD: Nonsense-mediated decay; RR: Regulatory region; SN: Synonymous; SPR: Splice Region; TFB: Transcription Factor Binding site; US: Upstream.

^3^Variant Analysis was performed with Ensembl's VEP (GRCh37.p13). Multiple type of variants are listed for a given gene when these variant occur in different transcripts.

^4^This SNP has now merged into a new SNP (GRCh38).

^5^For miRNA Gene Regions this column has the miRNA with the SNP; for miRNA-target genes this column has the associated miRNA for the mRNA with the SNP.

^6^Association seen in literature.

*Names in parentheses are alternatives.

**Table 3:** Associations of miRNA expression by SNPs in miRNA genes and miRNA-target genes between tumor and non-tumor tissues (adjusted for age, sex, and center).

			AA				AB				BB				p-values		
Gene(s)	SNP	miRNA	N	Mean	T % 0 Exp	N % 0 Exp	N	Mean	T % 0 Exp	N % 0 Exp	N	Mean	T % 0 Exp	N % 0 Exp	Raw	FDR	Variant Type^3^
**miRNA**																	
*MIR605*, *PRKG1*	rs2043556, 0 = TT 1 = TC 2 = CC	hsa-miR-605	259	-1.07	79.92	51.35	110	-0.36	75.45	61.82	14	0.53	64.29	57.14	0.007	0.36	RR. NC, NCE of MIR605. IN of PRKG1. US of RP11-539E19.2.
*MIR548AP*	rs2344843, 0 = AA 1 = AG 2 = GG	hsa-miR-548ap-5p	163	0.39	96.93	88.96	167	-0.42	98.80	85.03	53	-0.66	100.00	84.91	0.029	0.54	DS of MIR548AP.
*MIR146A*	rs2910164, 0 = GG 1 = GC 2 = CC	hsa-miR-146a-5p	218	3.62	26.61	27.52	146	-0.21	43.15	26.71	19	2.96	31.58	5.26	0.002	0.30	NC, NCE.
*MIR143*	rs353292, 0 = GG 1 = GA 2 = AA	hsa-miR-143-5p	105	4.46	88.57	96.19	204	-0.28	91.18	87.25	74	-0.22	94.59	87.84	0.019	0.54	US of MIR143. DS, NC, NCE of
		hsa-miR-145-3p	105	2.95	95.24	98.10	204	-0.44	96.57	93.14	74	-2.22	100.00	91.89	0.008	0.36	MIR143HG. UP of MIR145.
*MIR182*	rs76481776 (related SNP)^1^	hsa-miR-182-5p	311	2.16	88.10	100.00	72	3.22	97.22	100.00	0	NA	NA	NA	0.024	0.54	INTERGENIC VARIANT.
	rs2693737, 0 = AA 1 = AG 2 = GG																
*MIR143*	rs353293, 0 = CC 1 = CT 2 = TT	hsa-miR-143-5p	106	4.46	88.68	96.23	203	-0.28	91.13	87.19	74	-0.22	94.59	87.84	0.014	0.47	US of *MIR143*. DS, NC, NCE, IN of
		hsa-miR-145-3p	106	2.95	95.28	98.11	203	-0.44	96.55	93.10	74	-2.22	100.00	91.89	0.008	0.36	MIR143HG. UP of MIR145.
**miRNA-Target**																	
*CAMK1D*	rs10508445, 0 = AA 1 = AG 2 = GG	hsa-miR-4481	112	-9.41	0.00	0.00	193	-4.02	0.00	0.00	78	0.84	0.00	0.00	0.022	0.54	IN, NC.
*SLC10A7*	rs1057560, 0 = GG 1 = GA 2 = AA	hsa-miR-25-3p	104	13.75	3.85	13.46	189	14.00	4.23	11.11	90	12.33	10.00	6.67	0.007	0.36	RR. 3' UTR, DS.
*GRINL1A*	rs1062707, 0 = TT 1 = TC 2 = CC	hsa-miR-424-3p	226	13.46	0.00	0.44	131	9.62	0.00	0.00	26	7.90	0.00	0.00	0.025	0.54	IN, NC, DS, MND, 3' UTR, SN of *GCOM1* ^7^. 3' UTR, NMD, SN, IN,
		hsa-miR-424-5p	226	4.37	66.37	89.82	131	2.65	68.70	87.02	26	-0.05	76.92	88.46	0.022	0.54	NC, NCE of POLR2M7.
*KSR2*	rs11068503, 0 = TT 1 = TC 2 = CC	hsa-miR-149-3p	126	1.33	0.00	0.00	178	-1.79	0.00	0.00	79	-4.84	0.00	0.00	0.007	0.36	3' UTR, DS.
*EGFL7*, *LOC101928-612*	rs1332793, 0 = TT 1 = TC 2 = CC	hsa-miR-126-3p	151	0.87	6.62	9.27	183	-1.63	10.93	5.46	49	0.07	10.20	4.08	0.011	0.39	US, IN of *EGFL7*. US of *RP11-251M1*.*1*.
*VTCN1*	rs13505, 0 = AA 1 = AC 2 = CC	hsa-miR-196a-5p	207	1.66	42.03	25.12	147	2.52	40.82	27.89	29	3.51	17.24	27.59	0.036	0.64	RR. 3' UTR, DS.
*CD86*	rs17281995, 0 = GG 1 = GC 2 = CC	hsa-miR-212-3p	274	0.35	3.65	4.74	100	-0.53	9.00	8.00	9	-2.73	22.22	0.00	0.047	0.73	3’ UTR, DS.
*IL6ST*	rs2228043, 0 = GG 1 = GC 2 = CC	hsa-miR-221-3p	298	8.21	15.10	35.57	85	11.62	12.94	45.88	0	NA	NA	NA	0.027	0.54	3' UTR, US, NC, NCE, MS, NMD, IN.
*WWP2*	rs2270841, 0 = CC 1 = CT 2 = TT	hsa-miR-140-3p	199	-3.36	24.12	12.56	154	-1.51	18.83	11.69	30	-0.79	16.67	13.33	0.025	0.54	DS of *MIR140*. SN, US, NC, NCE, DS of *WWP2*.
*TAF1C*	rs2288024, 0 = TT 1 = TC 2 = CC	hsa-miR-103a-3p	358	14.46	3.07	0.84	25	22.63	0.00	0.00	0	NA	NA	NA	0.009	0.39	3' UTR, DS, IN, NC, NCE of *DNAAF1*. DS, 3' UTR, NC, NCE of *TAF1C*.
*TNFRSF4*	rs2298209, 0 = GG 1 = GC 2 = CC	hsa-miR-200b-3p	374	14.66	2.14	0.27	9	57.69	0.00	0.00	0	NA	NA	NA	0.043	0.70	DS, NC, NCE, 3’ UTR of *TNFRSF4*. UP of *TNFRSF18*.
*IL22RA2*	rs276466, 0 = AA 1 = AG 2 = GG	hsa-miR-30c-2-3p	212	0.47	9.43	13.21	149	-0.31	11.41	8.72	22	-1.07	22.73	4.55	0.001	0.22	3' UTR.
*ZNF396*	rs2909339, 0 = GG 1 = GA 2 = AA	hsa-miR-145-3p	267	-0.67	97.75	93.26	105	1.84	94.29	97.14	11	-0.22	100.00	90.91	0.038	0.64	DS, 3' UTR.
*PRKAR1A*	rs8905, 0 = TT 1 = TG 2 = GG	hsa-miR-214-3p	285	5.60	15.79	13.33	87	6.97	6.90	17.24	11	9.22	9.09	27.27	0.006	0.36	DS of *FAM20*. 3' UTR, DS, IN, NMD of *PRKR1A*.
*ZNF257*	rs9304994, 0 = AA 1 = AG 2 = GG	hsa-miR-557	135	8.03	0.00	0.00	183	-0.62	0.00	0.00	65	-2.80	0.00	0.00	0.029	0.54	3' UTR, NMD, DS.

^1^Related SNPs are those in linkage disequilibrium to SNPs within the dataset.

^2^EX: Exon; DS: Downstream; IN: intron; MS: Missense; NC: Non-coding; NCE: Non-coding exon; NMD: Nonsense-mediated decay; RR: Regulatory region; SN: Synonymous; SPR: Splice Region; TFB: Transcription Factor Binding site; US: Upstream.

^3^Variant Analysis was performed with Ensembl's VEP (GRCh37.p13). Multiple type of variants are listed for a given gene when these variant occur in different transcripts. Some additional genes not analyzed are listed by VEP.

^4^This SNP has now merged into a new SNP (GRCh38).

^5^For miRNA Gene Regions this column has the miRNA with the SNP; for miRNA-target genes this column has the associated miRNA for the mRNA with the SNP.

^6^Association seen in literature.

^7^Related genes/proteins of gene analyzed.

**Table 4:** MiRNAs significantly expressed between tumor and non-tumor colonic tissue that were identified with non-tumor and tumor/non-tumor SNP associations.

		Tumor		Normal		
miRNA	Associated Gene(s)	Mean	% 0 Expr.	Mean	% 0 Expr.	p-value
**Non-tumor**						
hsa-miR-1207-5p	*ICOS*	1832.06	0	2002.56	0	<0.01
hsa-miR-1307-3p	*MIR1307*, *USMG5*	11.04	0.4	12.87	0.1	<0.01
hsa-miR-1539	*RPL17*, *RPL17-C18orf32*, *SNORD58A*, *SNORD58*	3.21	48.1	3.09	41.2	0.43
hsa-miR-181a-5p	*SELS*	35.4	0.3	25.35	0.2	<0.01
hsa-miR-196b-5p	*ICOS*	15.72	23.2	6.04	22.3	<0.01
hsa-miR-19b-3p	*KIAA0423 (FAM179B)*	23.81	8.8	8.19	17.4	<0.01
hsa-miR-2117	*ICOS*, *CD80*	1.47	75.2	3.68	45.5	<0.01
hsa-miR-3196	*NKAIN4*, *FLJ16779*	1207.4	0	1373.03	0	<0.01
hsa-miR-3652	*GNN*, *HSP90B1*, *MIR3652*	149.06	0	158.9	0	<0.01
hsa-miR-4513	*CSK*	28.51	0	29.25	0	<0.01
hsa-miR-5196-5p	*CD22*, *MIR5196*	73.87	0	64.64	0	<0.01
hsa-miR-525-5p	*BRCA1*	1.87	56.1	2.38	46.6	<0.01
hsa-miR-630	*PARP1*	342.94	0	399.24	0	<0.01
hsa-miR-638	*DNM2*	3584.65	0	4091.42	0	<0.01
hsa-miR-648	*AFF1*	16.47	0	18.3	0	<0.01
hsa-miR-92b-3p	*FGF2*	0.99	89.1	0.6	89.1	<0.01
**Tumor/Non-Tumor Differential**						
hsa-miR-103a-3p	*TAF1C*	60.46	1.9	41.75	1	<0.01
hsa-miR-126-3p	*EGFL7*, *LOC101928612*	14.4	9.9	14.36	8.3	0.32
hsa-miR-140-3p	*WWP2*	5.29	21.6	7.35	15.3	<0.01
hsa-miR-145-3p	*MIR143*, *ZNF396*	1.05	98.1	1.67	94.2	0.04
hsa-miR-149-3p	*KSR2*	33.69	0	35.89	0	<0.01
hsa-miR-182-5p	*MIR182*	2.15	91.5	0.01	99.9	<0.01
hsa-miR-200b-3p	*TNFRSF4*	143.75	1.6	120.84	0.7	0.44
hsa-miR-212-3p	*CD86*	9.67	5	9.55	5	0.52
hsa-miR-214-3p	*PRKAR1A*	11.35	12.4	5.13	17	<0.01
hsa-miR-221-3p	*IL6ST*	11.57	17.6	2.62	40.3	<0.01
hsa-miR-30c-2-3p	*IL22RA2*	4.84	11.3	4.92	10.7	0.27
hsa-miR-424-3p	GRINL1A	36.22	0.2	24.48	0.1	<0.01
hsa-miR-424-5p	GRINL1A	3.97	69.2	0.53	89.5	<0.01
hsa-miR-4481	*CAMK1D*	78.1	0	84.13	0	<0.01
hsa-miR-548ap-5p	MIR548AP	0.83	97.8	0.96	86.5	<0.01
hsa-miR-557	ZNF257	74.59	0	74.44	0	0.34
hsa-miR-605	MIR605, PRKG1	1.54	79.2	2.03	59.1	<0.01
hsa-miR-196a-5p	VTCN1	6.27	39	3.66	31.9	<0.01
**Both Tumor/Non-tumor and Non-tumor**						
hsa-miR-143-5p	MIR143, NRSN1, LAD1	2.46	92.7	2.35	90.5	0.81
hsa-miR-146a-5p	MIR146A	8.45	33.1	5.69	28.3	0.01
hsa-miR-25-3p	MCM7, MIR106B, MIR25, MIR93, MCM7, AP4M1, SLC10A7	24.17	5.4	9.73	12.4	<0.01

## Results

Prior to adjustment for multiple comparisons, six SNPs within miRNA genes and 16 SNPs within miRNA-target genes were associated with differentially expressed miRNAs in non-tumor colonic tissue by genotype (Table 2). Twelve of the 16 miRNA-target gene SNPs were within the 3’ UTR. After adjustment for multiple comparisons, none of the identified associations remained statistically significant.

Six SNPs within miRNA genes and 15 SNPs within miRNA-targets genes were identified as being associated with differential miRNA levels between tumor and non-tumor tissue (Table 3); 12 of the 15 SNPs within miRNA-target genes occurred in the 3’ UTR. As was seen with the non-tumor associations, these miRNA-SNP associations were not significant after adjustment for multiple comparisons.

Further evaluation of miRNAs that were significantly influenced by SNPs prior to adjustment for multiple comparison in either non-tumor tissue or when comparing differences between tumor/non-tumor (see Tables 1 and 2 respectively) showed that all but seven miRNAs were significantly differentially expressed between colon tumor and non-tumor tissue (Table 4). Of those miRNAs associated with SNPs in non-tumor tissue (Table 2), nine miRNA were statistically significantly downregulated in tumor tissue and six were upregulated. Evaluation of miRNAs associated with SNPs in Table 3 (differentially expressed across genotype between tumor/non-tumor tissue types) showed that six were statistically significantly upregulated and six were downregulated in tumor tissue relative to non-tumor tissue. Two of the three miRNAs that were seen in both Tables 1 and 2, hsa-miR-146a-5p and hsa-miR-25-3p, were both statistically significantly upregulated in tumor tissue relative to non-tumor tissue (Table 4).

Evaluation of associations between colon cancer and SNPs that were significantly associated with miRNA expression levels showed two SNPs that were also significantly associated with increased risk of colon cancer (Table 5). Within the 3’ UTR of *BRCA1*, the homozygote variant genotype of rs8176318 was associated with increased risk of colon cancer (OR_AA_ 1.34 95% CI 1.01, 1.78. The associated miRNA, hsa-miR-525-5p, was not expressed in a large portion of the population with the homozygous-rare genotype; therefore, differences in expression across genotypes could be attributable to the reduction in the percent expressing. A second SNP, rs8905, within the 3’ UTR of *PRKAR1A* was associated with over a twofold increased risk of colon cancer (OR_GG_ 2.31 95% CI 1.11, 4.77). A third SNP, rs276466, was associated with increased risk of colon cancer with the heterozygote genotype (OR_AG_ 1.21 95% CI 1.02, 1.44) only and not the homozygote rare genotype and therefore may be a spurious association.

**Table 5:** Associations between SNPs associated with miRNAs in non-tumor and tumor/non-tumor differential expression and risk of colon cancer.

		AA		AB				BB			
SNP	Associated Gene(s)	Controls	Cases	Controls	Cases	OR	(95% CI)	Controls	Cases	OR	(95% CI)
**Non-tumor**											
rs10406069, 0 = GG 1 = GA 2 = AA	*CD22*, *MIR5196*	767	737	350	330	1	(0.80, 1.16)	56	48	0.9	(0.57, 1.28)
rs1053047, 0 = GG 1 = GA 2 = AA	*NRSN1*	321	323	591	544	0.9	(0.75, 1.11)	261	248	0.9	(0.74, 1.19)
rs1053667, 0 = TT 1 = TC 2 = CC	*KIAA0423*	1058	998	115	117	1.1	(0.83, 1.43)				
rs12232826, 0 = GG 1 = GT 2 = TT	*DNM2*	1087	1026	86	89	1.1	(0.81, 1.50)				
rs1378940, 0 = AA 1 = AC 2 = CC	*CSK*	502	501	520	476	0.9	(0.77, 1.09)	151	138	0.9	(0.70, 1.18)
rs1527423, 0 = AA 1 = AG 2 = GG	*MCM7*, *MIR106B*, *MIR25*, *MIR93*	332	299	574	557	1.1	(0.89, 1.31)	267	259	1.1	(0.86, 1.37)
rs1559931, 0 = GG 1 = GA 2 = AA	*ICOS*	659	652	441	395	0.9	(0.76, 1.07)	73	68	0.9	(0.65, 1.31)
rs16848494, 0 = CC 1 = CT 2 = TT	*LAD1*	1125	1063	48	52	1.1	(0.74, 1.65)				
rs17703261, 0 = AA 1 = AT 2 = TT	*AFF1*	776	711	353	369	1.1	(0.94, 1.35)	44	35	0.8	(0.53, 1.33)
rs17797090, 0 = GG 1 = GA 2 = AA	*GNN*, *HSP90B1*, *MIR3652*	981	939	192	176	0.9	(0.75, 1.18)				
rs1943676, 0 = AA 1 = AG 2 = GG	*RPL17*, *RPL17-C18orf32*, *SNORD58A*, *SNORD58*	506	499	526	498	1	(0.80, 1.14)	141	118	0.9	(0.66, 1.14)
rs4404254, 0 = TT 1 = TC 2 = CC	*ICOS*	658	652	442	395	0.9	(0.75, 1.07)	73	68	0.9	(0.65, 1.31)
rs720607, 0 = GG 1 = GA 2 = AA	*NKAIN4*, *FLJ16779*	362	364	574	547	1	(0.79, 1.15)	237	204	0.9	(0.67, 1.08)
rs7628626, 0 = CC 1 = CA 2 = AA	*CD80*	805	726	336	351	1.2	(0.97, 1.40)	32	38	1.3	(0.80, 2.10)
rs7683093, 0 = CC 1 = CG 2 = GG	*FGF2*	856	823	291	265	1	(0.78, 1.15)	26	27	1.1	(0.61, 1.84)
rs7911488, 0 = AA 1 = AG 2 = GG	*MIR1307*, *USMG5*	533	504	524	491	1	(0.83, 1.17)	116	120	1.1	(0.83, 1.46)
**rs8176318, 0 = CC 1 = CA 2 = AA**	***BRCA1***	**560**	**484**	**504**	**504**	**1.2**	**(0.96, 1.36)**	**109**	**127**	**1.3**	**(1.01, 1.78)**
rs8679, 0 = AA 1 = AG 2 = GG	*PARP1*	704	684	414	373	0.9	(0.78, 1.11)	55	58	1.1	(0.73, 1.58)
rs9874, 0 = TT 1 = TC 2 = CC	*SELS*	885	837	265	255	1	(0.83, 1.24)	23	23	1.1	(0.60, 1.95)
rs999885, 0 = AA 1 = AG 2 = GG	*MCM7*, *AP4M1*	327	298	577	555	1.1	(0.87, 1.28)	269	262	1.1	(0.86, 1.36)
**Tumor/Non-tumor**											
rs10508445, 0 = AA 1 = AG 2 = GG	*CAMK1D*	307	303	566	555	1	(0.82, 1.22)	300	257	0.9	(0.70, 1.11)
rs1062707, 0 = TT 1 = TC 2 = CC	*GRINL1A*	680	671	414	379	0.9	(0.78, 1.10)	79	65	0.8	(0.58, 1.16)
rs11068503, 0 = TT 1 = TC 2 = CC	*KSR2*	366	339	555	544	1.1	(0.88, 1.29)	252	232	1	(0.80, 1.27)
rs1332793, 0 = TT 1 = TC 2 = CC	*EGFL7*, *LOC101928612*	462	433	542	522	1	(0.86, 1.23)	169	160	1	(0.77, 1.28)
rs13505, 0 = AA 1 = AC 2 = CC	*VTCN1*	623	609	450	427	1	(0.81, 1.15)	100	79	0.8	(0.59, 1.12)
rs17281995, 0 = GG 1 = GC 2 = CC	*CD86*	848	809	304	277	1	(0.80, 1.17)	21	29	1.4	(0.79, 2.48)
rs2043556, 0 = TT 1 = TC 2 = CC	*MIR605*, *PRKG1*	737	707	374	366	1	(0.84, 1.21)	62	42	0.7	(0.48, 1.08)
rs2228043, 0 = GG 1 = GC 2 = CC	*IL6ST*	898	841	275	274	1.1	(0.88, 1.29)				
rs2270841, 0 = CC 1 = CT 2 = TT	*WWP2*	637	577	454	445	1.1	(0.91, 1.28)	82	93	1.3	(0.92, 1.75)
rs2288024, 0 = TT 1 = TC 2 = CC	*TAF1C*	1080	1046	93	69	0.8	(0.55, 1.05)				
rs2298209, 0 = GG 1 = GC 2 = CC	*TNFRSF4*	1132	1088	41	27	0.7	(0.41, 1.10)				
rs2344843, 0 = AA 1 = AG 2 = GG	*MIR548AP*	472	467	529	499	1	(0.81, 1.16)	172	149	0.9	(0.67, 1.12)
rs2693737, 0 = AA 1 = AG 2 = GG	*MIR182*	968	913	205	202	1	(0.84, 1.29)				
**rs276466, 0 = AA 1 = AG 2 = GG**	***IL22RA2***	**720**	**642**	**385**	**419**	**1.2**	**(1.02, 1.44)**	68	54	0.9	(0.61, 1.29)
rs2909339, 0 = GG 1 = GA 2 = AA	*ZNF396*	871	792	278	295	1.2	(0.97, 1.42)	24	28	1.3	(0.73, 2.23)
rs353292, 0 = GG 1 = GA 2 = AA	*MIR143*	341	309	609	573	1.1	(0.86, 1.27)	223	233	1.2	(0.92, 1.49)
rs353293, 0 = CC 1 = CT 2 = TT	*MIR143*	340	310	610	572	1	(0.85, 1.25)	223	233	1.2	(0.91, 1.48)
**rs8905, 0 = TT 1 = TG 2 = GG**	***PRKAR1A***	**928**	**836**	**234**	**256**	**1.2**	**(0.98, 1.46)**	**11**	**23**	**2.3**	**(1.11, 4.77)**
rs9304994, 0 = AA 1 = AG 2 = GG	*ZNF257*	380	386	599	534	0.9	(0.73, 1.05)	194	195	1	(0.78, 1.27)
**Both Tumor/Non-tumor and Non-tumor**											
rs1057560, 0 = GG 1 = GA 2 = AA	*SLC10A7*	316	308	590	555	1	(0.79, 1.17)	267	252	1	(0.77, 1.23)
rs2910164, 0 = GG 1 = GC 2 = CC	*MIR146A*	665	652	439	405	0.9	(0.78, 1.11)	69	58	0.9	(0.59, 1.23)

^1^Referent group is AA (homozygote common genotype); AB (heterozygote genotype); BB (homozygote rare genotype).

^2^Odds Ratios (OR) and 95% Confidence Intervals (CI) adjusted for age, sex and center.

**Bolded** items are significantly associated with risk of colon cancer.

## Discussion

It has been suggested that SNPs associated with risk of colon cancer could be functioning through their impact on miRNA regulation [9]. In this study, we investigated SNPs within miRNA gene and miRNA-target gene regions previously reported in the literature as associated with cancer, to evaluate if these SNPs influence miRNA expression and alter colon cancer risk. Of the 327 SNP/miRNA pairs evaluated, 22 SNPs were associated with significant (P<0.05) differences in miRNA expression in non-tumor tissue by genotype, and 21 SNPs were associated with significant differential miRNA expression between tumor and non-tumor tissue by genotype. Evaluation of these SNPs with colon cancer showed only two SNPs were significantly associated with colon cancer risk. This suggests that the majority of the hypothesized associations are not supported when evaluated directly with miRNA data.

Two SNPs in miRNA-target genes were identified as associated with an increased risk of colon cancer; these SNPs also were associated with statistically significant mean differential miRNA expression across genotypes. Of these, rs8176318, located in the 3’ UTR of *BRCA1* and predicted to be a binding site for hsa-miR-525-5p when the G allele (or C in our data) is present [21], was associated with a linear decrease in hsa-miR-525-5p expression in non-tumor tissue when comparing homozygote common (CC) to homozygote rare genotypes (AA). MiRNA hsa-miR-525-5p also was downregulated in tumor as compared to non-tumor tissue (p = 0.0044) and rs8176318 was significantly associated with an increase in risk of colon cancer (OR_AA_ 1.34 95% CI 1.01, 1.78). This variant has been reported as being associated with decreased *BRCA1* expression, increased breast cancer risk, and greater likelihood of having stage IV breast cancer [56]. Expression levels of this miRNA are somewhat low, and may therefore less precise, however, together these findings could support the claim that the SNP rs8176318 contributes to the incidence and progression of some breast and colon cancers through altered miRNA regulation within these tissues.

We also identified another SNP, rs8905, in the 3’ UTR of *PRKAR1A*, that was significantly associated with decreased expression in tumors relative to non-tumor tissue of the miRNA that targets *PRKAR1A*, hsa-miR-214-3p, in subjects of the homozygote common (TT) genotype as compared to the heterozygote and homozygote rare (TG, GG respectively) genotypes. Additionally, a much higher percentage of non-tumor tissue did not express hsa-miR-214-3p in the heterozygote genotype as compared to the TT (common) genotype, and even less expression was observed for the homozygote rare genotype. Hsa-miR-214-3p was statistically significantly upregulated in tumor versus non-tumor tissues, and the SNP rs8905 was seen to significantly increase the risk of colon cancer (OR_GG_ 2.31 95% CI 1.11, 4.77). In this instance, it is likely that the alteration of the miRNA expression is caused by the SNP and this directly contributes to colon tumor risk.

Our data did support some of the literature in terms of SNP and miRNA associations. In a recent study, two SNPs within the promoter region of hsa-miR-143/145, rs353292 and rs353293, were significantly associated with an increased risk of colorectal cancer (CRC) in a predominately Chinese population [9]. As these SNPs are in the promoter region of miRNAs, Li et al. proposed that the mechanism by which these SNPs increased CRC risk was through the expression of hsa-miR143/145 [9]. In our investigation, both rs353292 and rs353293 were associated with differential expression of hsa-miR-145-3p and hsa-miR-143-5p between genotypes between tumor and non-tumor tissues (Table 3). In both instances, miRNA expression was greater among individuals with two copies of the common allele (i.e. homozygous common genotype) in non-tumor tissue. Having a copy of the variant allele resulted in a decrease in miRNA expression in tumor tissue compared to non-tumor tissue. Of these two, hsa-miR-145-3p was significantly differentially expressed between tumor and non-tumor tissue, however neither of these SNPs were significantly associated with colon cancer.

Dikaiakos et al. [44] reported higher incidences of CRC among individuals with the CC genotype and with the C allele of rs2910164, which is in the precursor miRNA region of hsa-miR-146a, and suggested that this increased risk is attributed to alterations in the miRNA expression and alterations to its structure [44]. We observed that miR-146a is differentially expressed across genotypes of rs2910164 for non-tumor tissue as well as between tumor and non-tumor tissues. Additionally, expression of hsa-miR-146a-5p was statistically significantly upregulated in tumor tissue compared to non-tumor tissue, suggesting that this SNP may impact miRNA expression. However, unlike Dikaiakos, we did not observe that this SNP was associated with colon cancer.

In a review by Ryan et al. [20], *AFF1* rs17703261 and *KIAA0423* (or *FAM179B*) rs1053667, were significantly associated with non-specified cancer risk. In that work, *AFF1* rs17703261 was inversely associated with cancer risk (OR for the A/T was 0.34 95% CI 0.20, 0.58) and *KIAA0423* rs1053667 was directly associated with cancer risk (OR for the C/T was 3.29 95% CI 1.72, 6.32) [20]. The miRNAs that target *AFF1* are hsa-miRs-19a, -19b, -585, and -648. In our study, we identified a significant decrease in expression of hsa-miR-648 in the homozygote rare (TT) genotype in non-tumor tissue. Additionally, we found that expression of this miRNA is significantly downregulated in tumor tissue compared to non-tumor tissues. However, we did not observe an association between *AFF1* rs17703261 with the risk of developing colon cancer. The miRNAs that were identified as targeting *KIAA0423* in Ryan et al. were hsa-miRs-19a and -19b. We identified significantly lower expression of hsa-miR-19b-3p in the heterozygote genotype as compared to the homozygote common genotype in non-tumor colonic tissues. We also found expression of this miRNA to be upregulated in tumor versus non-tumor tissues. However, rs1053667 was not associated with risk of colon cancer.

Wu et al. [43] investigated several SNPs within the 3’ UTRs of genes in the B7/CD28 signaling pathway, involved in T cell stimulation, including rs7628626 (*CD80*), rs1305 (*VTCN1*), and rs44044254 and rs1559931 (*ICOS*). In their study, they found an increased risk of CRC with the homozygote rare genotype of rs1305 and a decreased risk of CRC with the codominant and dominant model for rs4404254 and rs1559931. We saw slightly reduced, non-significant risk for all of these SNPs with colon cancer.

It has been suggested that miRNA expression levels are associated with expression of their corresponding target genes, and that the presence of the target genes prevents miRNA degradation by nucleases [11]. To investigate this relationship, we used miRNASNP v2.0 to evaluate SNPs within 3’ UTRs of mRNAs for changes in predicted miRNA regulation. Many of the SNPs discussed previously had documented predicted loss or gain of miRNA targeting based on the changes the SNP makes in the 3’ UTR of the target mRNA, and thus the binding region of the miRNA. An example of this change is the SNP in *AFF1*, rs17703261 which is predicted to cause a loss of targeting by hsa-miR-648. This miRNA is slightly downregulated in tumor as compared to non-tumor tissues in our data. The homozygous rare genotype has significantly less expression of hsa-miR-648 than the other genotypes. Other examples are *SLC10A7* rs105760 and *KIAA0423* rs1053667, which are predicted to cause loss of targeting by hsa-miR-25-3p and hsa-miR-19b-3p, respectively. Rs1053667 and rs105760 were associated with reduced expression in tumor as compared to non-tumor tissues of the predicted lost miRNAs with the variant allele. A further example, rs9874 (*SELS)* is predicted to cause a gained targeting by hsa-miR-181a-5p, and we see an increase in mean expression between tumor and non-tumor tissue with the variant allele. Additionally, *BRCA1* rs8176318, is predicted to have a gain of function for hsa-miR-20a-3p. We evaluated this miRNA/SNP pair and did not see an association between the predicted gained miRNA and this SNP. However, as mentioned previously, *BRCA1* rs8176318 was associated with the G allele specific (C in our tables) miRNA hsa-miR-525-5p and we did see a reduction in expression of hsa-miR-525-5p in non-tumor tissues with the variant allele as well as a lowered level of mean miRNA expression in tumor tissue as compared to non-tumor tissue. These results would support the theory that a loss of the presence of the target (as a result of the SNP) causes a reduction in the level of miRNA expression.

There are several considerations when evaluating our results. First, our study is comprised of colon cancer cases while many studies included both colon and rectal cancer cases. Differences in associations with cancer could differ based on the differences in cases definition. Our findings elucidate the complexity of miRNA regulation, especially the role of SNPs in the carcinogenic process that involves miRNA. MiRNAs are involved in the regulation of multiple genes and, even though SNPs were associated with miRNAs, the influence of miRNAs on other genes may determine the association with cancer. For example, hsa-miRNA-25-3p, along with its regulation of *SLC10A7*, is associated with *MCM7*, *MIR106B*, *MIR25*, *MIR93* and *AP4M1* and several SNPs, rs105756, rs999885, and rs1527423. This can make the interpretation of results difficult. While there are study limitations, a major strength of this study is the ability to evaluate hypothesized SNP/miRNA associations and to evaluate associated SNPs and miRNAs with colon cancer.

Out of the 327 unique SNP-miRNA pairs evaluated only 41 were associated with miRNA expression levels. Of these, only two SNPs were associated with colon cancer risk. While it has been hypothesized that SNPs in miRNA regions can influence cancer risk through miRNA regulation, our data suggest that such associations are relatively rare when evaluating colon cancer.
